# Immunologic Difference between Hypersensitivity to Mosquito Bite and Hemophagocytic Lymphohistiocytosis Associated with Epstein-Barr Virus Infection

**DOI:** 10.1371/journal.pone.0076711

**Published:** 2013-10-18

**Authors:** Wen-I Lee, Jainn-Jim Lin, Meng-Ying Hsieh, Syh-Jae Lin, Tang-Her Jaing, Shih-Hsiang Chen, Iou-Jih Hung, Chao-Ping Yang, Chin-Jung Chen, Yhu-Chering Huang, Shin-Pai Li, Jing-Long Huang

**Affiliations:** 1 Primary Immunodeficiency Care And Research (PICAR) Institute, Chang Gung Children’s Hospital, Taoyuan, Taiwan; 2 Department of Pediatrics, Division of Allergy Asthma and Rheumatology, Chang Gung Children’s Hospital, Chang Gung University College of Medicine, Taoyuan, Taiwan; 3 Graduate Institute of Medical Clinics, Chang Gung University College of Medicine, Taoyuan, Taiwan; 4 Department of Pediatrics, Division of Neurology, Chang Gung Children’s Hospital, Taoyuan, Taiwan; 5 Department Pediatrics, Division of Hematology/Oncology, Chang Gung Children’s Hospital, Taoyuan, Taiwan; 6 Department of Pediatrics, Division of Critical Care and Emergency Medicine, Chang Gung Children’s Hospital, Taoyuan, Taiwan; 7 Department of Pediatrics, Division of Infection, Chang Gung Children’s Hospital, Taoyuan, Taiwan; 8 Department of Microbiology and Immunology, Chang Gung University College of Medicine, Taoyuan, Taiwan; Duke University Medical Center, United States of America

## Abstract

Hemophagocytic lymphohistiocytosis (HLH) is a life-threatening, virus-triggered immune disease. Hypersensitivity to mosquito bite (HMB), a presentation of Chronic Active Epstein-Barr Virus infection (CAEBV), may progress to HLH. This study aimed to investigate the immunologic difference between the HMB episodes and the HLH episodes associated with EBV infection. Immunologic changes of immunoglobulins, lymphocyte subsets, cytotoxicity, intracellular perforin and granzyme expressions, EBV virus load and known candidate genes for hereditary HLH were evaluated and compared. In 12 HLH episodes (12 patients) and 14 HMB episodes (4 patients), there were both decreased percentages of CD4+ and CD8+ and increased memory CD4+ and activated (CD2+HLADR+) lymphocytes. In contrast to HMB episodes that had higher IgE levels and EBV virus load predominantly in NK cells, those HLH episodes with virus load predominantly in CD3+ lymphocyte had decreased perforin expression and cytotoxicity that were recovered in the convalescence period. However, there was neither significant difference of total virus load in these episodes nor candidate genetic mutations responsible for hereditary HLH. In conclusion, decreased perforin expression in the HLH episodes with predominant-CD3+ EBV virus load is distinct from those HMB episodes with predominant-NK EBV virus load. Whether the presence of non-elevated memory CD4+ cells or activated lymphocytes (CD2+HLADR+) increases the mortality rate in the HLH episodes remains to be further warranted through larger-scale studies.

## Introduction

The Epstein-Barr virus (EBV) infects B cells through surface CD21 in healthy individuals who are often asymptomatic or may present as infectious mononucleosis (IM) [1]. The outgrowth of EBV-infected B cells is controlled by T help cells secreting interferon (IFN)-γ and NK-mediated cytoxicity, and later destroyed by EBV-specific cytotoxic T lymphocytes [2], [3]. Patients with chronic active EBV (CAEBV) infection may have IM-like chronic symptoms such as fever and lymphadenopathy, and serologic evidence of persistent EBV infection [4]–[9]. Moreover, CAEBV can be exacerbated into fulminant (catastrophic) hemophagocytic lymphohistiocytosis (HLH) [10]–[14] and present with cytopenia, coagulopathy, central nervous system symptoms, and lipid changes, aside from IM-like features [15]. Known candidate mutations of *SH2D1A/SAP*, *PRF1*, *UNC13D*, *STX11, STXBP2*, *XIAP*, and *ITK* can inhibit the exocytotic process of polarization, docking, priming, and fusion in cytotoxic T/natural killer (NK) cells, subsequently lead to defective cytotoxicity and overwhelming HLH in some rare hereditary and sporadic cases [16]–[20].

Hypersensitivity to mosquito bite (HMB) is a unique feature characterized by bulla formation with intense erythema on mosquito-bitten sites, escar healing and systemic manifestations like fever, lymphadenopathy, and splenomegaly [21], [22]. Around 70% of CAEBV patients present as the HMB episode (HMB-CAEBV) and have the potential of developing fulminant HLH [23].

To understand the possible mechanisms of HMB transformation into fulminant HLH, we evaluated and compared immunologic changes of immunoglobulins, lymphocyte subsets, cytotoxicity, intracellular perforin and granzyme expressions, EBV virus load and known candidate genes in patients with the episodes of HMB-CAEBV and EBV-HLH.

## Results

### Patients’ Characteristics

**Figure 1:**
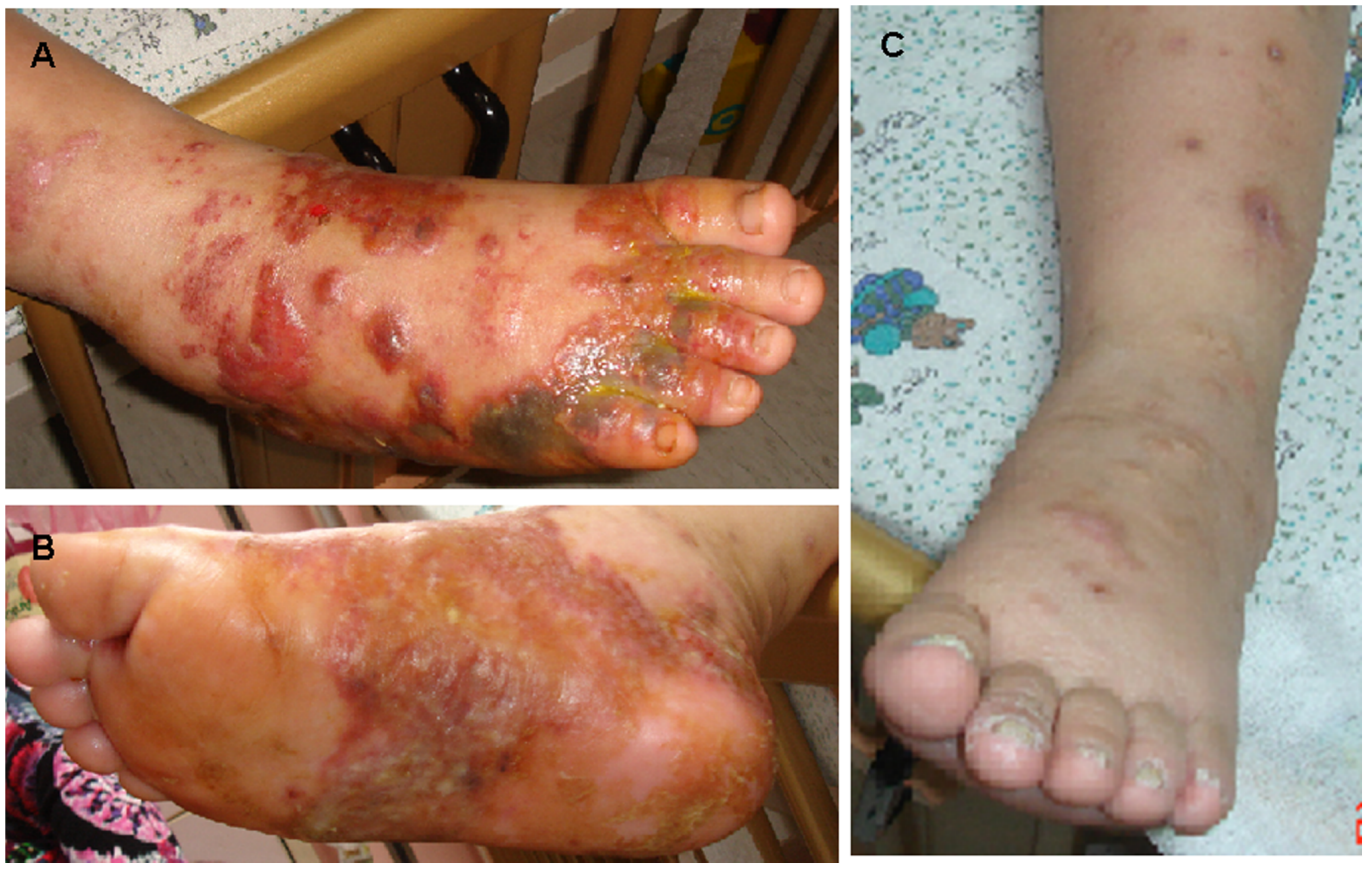
There were clear and hemorrhagic bulla with intense erythematous swelling at mosquito-bitten sites on the (A) right leg dorsum and (B) palm. Necrosis and ulcers clustered on the base of the toes and turned into escar formation after recovery. (**C**) Previous escar scar remitted and centrally dipped like a volcano on the left.

During the 20-year period of 1993–2012, fourteen HMB episodes in 4 CAEBV patients (one female) and twelve HLH episodes in 12 patients (five females) associated with EBV infection (EBV-HLH) were studied in Table 1. The HMB episode could be a characteristic feature of CAEBV along with fever, lymphadenopathy or/and hepatosplenomagaly. At mosquito-bitten sites (Fig. 1), clear or/and hemorrhagic bulla with intense erythematous swelling typically occurred. They progressed into necrosis or ulcers, and healed with residual scarring as escar.

**Table 1:** Laboratory hematology, treatment, and prognosis of patients with hemophagocytic lymphohistiocytosis (HLH)* and hypersensitivity to mosquito bite (HMB) episodes related to EBV infection.

Patient/Sex	Onset Age (y)	Onset year(AD)	Fever	Cytopenia	Hemophagocytosis	Hypertriglycemiaand/orhypofibrinogenemia	Splenomegaly/Lymphadenopathy	Ferritin*	DecreasedNKactivity	AST/ALT*	Treatment	Deceasedaftertreatment	
**EBV-associated HLH (survival)**													
ES1/M	6Y2M	1995	+	+ (Hb = 8.7; PL = 12K)	+	+ (TG = 332)/−	+/+	+ (1479)	+	326/118	IVIG/ST/VP16		
ES2/F	3Y6M	1998	+	+ (Hb = 8.4; PL = 24K)	+	ND/ND	+/−	+ (3298)	ND	762/170	IVIG/ST		
ES3/M	10Y7M	2001	+	+ (Hb = 7.8; PL = 35K)	+	ND/ND	+/+	+ (11788)	ND	16/10	ST		
ES4/M	1Y	2005	+	+ (Hb = 8.2; PL = 20K)	+	+ (TG = 293)/ −	+/−	+ (13906)	+	603/358	IVIG/ST/CsA/VP16		
ES5/F	2Y10M	2006	+	+ (Hb = 8.3; Neu = 990; PL = 90K)	+	−/ND	+/−	+ (751)	+	2127/1665	IVIG/ST/CsA		
ES6^/^F	6Y6M	2006	+	+ (Hb = 8.5; PL = 56K)	+	−/−	+/−	+ (2404)	+	342/214	IVIG/ST/CsA/VP16		
**EBV-associated HLH (mortality)**													
EM1/M	3Y2M	1992	+	+ (Hb = 7.0; PL = 26 K)	+	ND/ND	+/+	+ (7821)	ND	471/204	IVIG/ST	14 days	
EM2/M	6Y6M	1993	+	+ (Hb = 5.5; Neu = 384; PL = 93K)	+	−/+ (Fibri = 80)	+/+	+ (2134)	ND	2898/1285	IVIG	8 days	
EM3/M	1Y10M	2001	+	+ (Hb = 7.9; Neu = 540; PL = 14K)	+	+ (TG = 295)/ND	+/−	+ (14523)	+	358/253	IVIG/ST/G-CSF	29 days	
EM4/F	1Y7M	2002	+	+ (Hb = 5.1; Neu = 36; PL = 70K)	+	+ (TG = 473)/+ (Fibri = 47)	+/+	+ (1543)	+	2109/485	IVIG	13 days	
EM5/F	5Y2M	2005	+	+ (Hb = 8.4; PL = 49K)	+	+ (TG = 506)/ND	+/+	− (334)	+	63/23	IVIG/ST	58 days	
EM6/M	11Y	2005	+	+ (Hb = 8.3; Neu = 60; PL = 67K)	+	−/+ (Fibri = 89)	+/−	− (345)	+	66/45	IVIG/CsA	15 days	
**Hypersensitivity to Mosquito bite (HMB)-CAEBV**													
H1/M	12Y3M	2003	+	−	−	−/ND	−/−	+ (2785)	−	237/174	ST/NSAID		
		2005	+	−	−	ND/ND	+/+	−	−	149/82	ST/NSAID		
		2006	+	−	−	ND/ND	+/+	−	−	73/65	ST/NSAID		
		2008	+	−	−	ND/ND	+/+	−	−	104/86	ST/NSAID		
		2011	+	−	−	−/−	+/+	−	−	54/67	ST/NSAID		
H2/M	4M	2005	+	−	−	−/−	−/−	+ (3479)	−	122/87	ST/NSAID		
		2007	+	−	−	−/−	+/+	−	−	64/43	ST/NSAID		
		2012	+	−	−	−/−	+/+	−	−	123/72	ST/NSAID		
H3/M	12Y	2005	+	−	−	ND/−	−/−	−	−	89/55	ST/NSAID		
		2006	+	−	−	ND/ND	+/+	−	−	75/58	ST/NSAID		
		2011	+	−	−	−/−	+/+	−	−	45/78	ST/NSAID		
H4/M	18 Y	2006	+	−	−	−/ND	−/−	−	−	114/62	ST/NSAID		
		2010	+	−	−	−/−	−/−	−	−	112/57	ST/NSAID		
		2012	+	−	−	−/−	+/+	−	−	137/69	ST/NSAID		

Abbreviations: M, male; F, female; Hb, hemoglobin; PL, platelet; Neu, neutrophil; TG, triglyceride; Fribi; fibrinogen; AST, aspartate aminotransferase; ALT, alanine aminotransferase; IVIG, intravenous immunoglobulin; ST, steroid prednisolone or dexamethasone; CsA, cyclosporine A; VP-16, etoposide; ND, not done; NSAID, Non-steroidal anti-inflammatory drug; CAEBV, Chronic active EBV infection.

* The diagnosis criteria included fever, splenomegaly, cytopenia (affecting 2 of 3 lineages, Hb <9 mg/dL; PL <100 K; and Neu <1000), hyper-triglycerides (265 mg/dL) or hypo-fibrinogenemia (1.5 g/L), hemophagocytosis, lower or abscent NK-cell activity, ferritin >500 ul/L and soluble CD25>2400 U/ml. Twelve patients with HLH reached at least 5 criteria without detectable soluble CD25.

a The normal range of fibrinogen, TG, ferritin, AST, and ALT was 190–380 mg/dL, <150 mg/ml, 10–322 ng/ml, 13–40 U/L, and <36 U/L, respectively.

In contrast to HMB-CAEBV episodes (the range of onset-age, 4 months-21 years; median, 12 years 3 months), acute EBV-HLH episodes (range, 1–11 years; median, 3 years 4 months) had cytopenia (Hb <9.0 mg/dl and thrombocytopenia <100,000/mm^3^ in all; neutropenia <1,000/mm^3^ in 5 patients), coagulopathy (abnormal PT, aPTT, D-dimmer, or fibrogen in 7 patinets), and atypical lymphocytes (over 10% in 3 patients). Both groups often had splenomegaly, lymphadenopathy, and varying degrees of elevated aspartate aminotransferase (AST) and/or alanine aminotransferase (ALT) levels.

The main treatment regimens in patients with HLH episodes based on the HLH 2004 guidelines [15] included IVIG, steroids (prednisolone or dexamethasone), etoposide, and cyclosporine A. Six (50%) of 12 acute EBV-HLH patients who did not receive etoposide (VP16) and cyclosporine A treatment were mortalities. In four HMB-CAEBV patients who did not develop HLH episode to date, NSAID or steroids were given for febrile episodes.

Serology studies for EBV in 26 episodes from 16 patients showed that 11 HLH patients (ES1-ES6 except ES4 and EM1-EM6) had primary EBV infection with positive anti-VCA-IgM or/and positive anti-EBEA (≥160) (Table 2). One episode of HLH (patient ES4) and 14 HMB episodes (4 patients H1–H4) had mainly positive anti-VCA IgG and/or negative VCA IgM, suggestive of EBV reactivation. The EBV viral load detected by copy numbers in all episodes was ≥10^2.5^ copies/ug, compatible with EBV activation [24]. In contrast to the HMB episodes with EBV copy number predominantly in NK cells, the HLH episodes had EBV virus load predominantly in lymphocytes (CD3+). Nonetheless, there was no significant difference in virus load of total lymphocytes among the HLH (survivors and fatal victims) and HMB episodes.

**Table 2:** EBV serology and evidence in EBV-HLH and HMB-CAEBV episodes.

Patient (Year)	Serum antibodies of EBV profile				EBV Virus load log copies/ug genomic DNA	
	EBEA	EBNA	VCA IgG	VCA IgM	CD3+	CD16+CD56+
EBV-HLH-Survival						
ES1 (1995)	**320**	**+**	**320**	−	**5.6**	NA
ES2 (1998)	NA	NA	**320**	**+**	**3.9**	2.8
ES3 (2001)	NA	NA	**320**	**+**	**4.7**	3.2
ES4 (2005)	**80**	**+**	**640**	−	**4.2**	2.3
ES5 (2006)	**80**	**+**	**320**	**+**	**3.7**	2.7
ES6 (2006)	**80**	−	**320**	**+**	**4.9**	2.4
EBV-HLH-Mortality						
EM1 (1992)	**320**	**+**	**1280**	+	NA	NA
EM2 (1993)	**160**	**+**	**160**	−	**4.6**	NA
EM3 (2001)	NA	**+**	**160**	**+**	**5.1**	NA
EM4 (2002)	20	NA	**NA**	**+**	**3.7**	3.0
EM5 (2005)	20	−	**320**	**+**	**4.5**	3.2
EM6 (2005)	**160**	**+**	**80**	−	**3.4**	2.1
HMB-CAEBV						
H1 (2003)	**80**	20	**640**	−	3.1	**5.2**
(2005)	**80**	**+**	**640**	−	4.1	**5.7**
(2006)	**160**	**+**	**320**	−	NA	NA
(2008)	20	**+**	**640**	−	2.7	**4.9**
(2011)	20	−	**1280**	−	3.0	**4.7**
H2 (2005)	**NA**	**+**	**−**	+	2.4	**5.1**
(2007)	20	**+**	**320**	−	NA	NA
(2012)	20	**+**	**640**	−	3.9	**4.7**
H3 (2005)	**160**	**+**	**−**	+	2.9	**3.7**
(2006)	**80**	**+**	**640**	−	NA	NA
(2011)	**80**	**+**	**1280**	−	2.7	**5.4**
H4 (2006)	20	**+**	**640**	−	2.9	**3.5**
(2010)	20	**+**	**160**	−	NA	NA
(2012)	20	**+**	**640**	−	3.6	**4.8**

Abbreviations: EBV, Epstein-Barr virus; ENEA, Epstein-Barr virus early antigen; EBNA, Epstein-Barr virus nuclear antigen; VCA, viral capsid antigen; IgG, immunoglobulin G; IgM, immunoglobulin M; NA, not available.

### Immunoglobulin and Lymphocyte Sub-population

In eight HLH episodes (8 patients) and 14 HMB episodes (4 patients), the basic immune function of immunoglobulins, lymphocyte sub-populations, memory cells, and activated lymphocytes revealed immune heterogeneity in each group. Based on the normal reference [25], relatively higher IgG, and/or IgA were present in two fatal HLH cases (patients EM3 and EM6), but not in HMB cases. The Trend of decreased percentages of total CD4+ but increased memory CD4+ and activated lymphocyte (CD2+HLADR+) was noted among survivors of the HLH and the HMB groups (Table 3).

**Table 3:** Serum immunoglobulin values and lymphocyte subsets in HLH-EBV and HMB-CAEBV episodes related to EBV infection.

Patient			Immunoglobulin level (mg/dl)^a^							Absolute lymphocyte count		Lymphocyte subsets percentages (%)^b^														
			IgM	IgA		IgG		IgE				CD4		CD8		CD19		CD16/56		Memory cell*					Activated lymphocyte	
																				CD4+T−			B−			
**EBV-associated survival**																										
ES1		1995	164	173		1190		79		1432		34.2		18.4		13.2		8.9		31.6		7.8			43.1	↑
ES4		2005	38	17		674		107	↑	2135		9.5	↓	14.8		71.5	↑	3.8		51.6	↑	1.4		↓	23.7	
ES5		2006	42	59		867		42		4290		19.0	↓	11.2	↓	9.1		56.9	↑	42.6	↑	3.6			65.1	↑
ES6		2006	143	69		1495		147	↑	3192		38.8		34.1		11.2		8		45.7	↑	18.9			34.1	
**EBV-associated dead**																										
EM3		2001	42	32		1975	↑	97		1547		32.4		18.5		11.2		10.8		9.4		5.4			18.9	
EM4		2002	32	46		568		56		446	↓	41.5		32.7		8.7		3.4		13.0		6.7			24.4	
EM5		2005	53	57		756		86		877		40.8		20.4		10.4		4.2		8.3		4.3			20.5	
EM6		2007	45	576	↑	2640	↑	92		340	↓	39.7		29.4		9.6		1.8	↓	10.2		2.3		↓	14.9	
**Hypersensitivity to Mosquito bite (HMB)**																										
H1	2003		110	236		1650	↑	1804	↑	1874		14.9	↓	10.9	↓	11.5		59.0	↑	48.2	↑	9.1			48.1	↑
	2005		89	215		1756	↑	1124	↑	1945		19.4	↓	11.4	↓	14.8		47.2	↑	54.2	↑	10.2			47.5	↑
	2006		142	198		1324		2468	↑	2147		22.4	↓	8.8	↓	21.0		39.6	↑	44.5	↑	16.5			61.4	↑
	2008		127	246		1942	↑	2497	↑	1258		27.3	↓	11.2	↓	19.7		38.7	↑	39.7	↑	11.8			50.9	↑
	2011		169	231		1237		1785	↑	2013		33.7		12.1	↓	17.5		45.2	↑	47.1	↑	14.9			49.1	↑
H2	2005		78	55		573		129	↑	4984	↑	40.9		19.5		18.0		11.4		27.5		7.2			14.3	
	2007		NA	NA		NA		NA		3278		24.2	↓	10.5		8.6		21.2	↑	34.2		14.2			32.5	
	2012		102	119		1745	↑	952	↑	2846		32.5		11.4		9.7		24.8	↑	39.5	↑	19.4			24.1	
H3	2005		271	228		1420	↑	1420	↑	3945		21.7	↓	13.7		5.4	↓	59.4	↑	46.8	↑	20.3		↑	66.1	↑
	2006		NA	NA		NA		NA		3125		24.5	↓	17.9		10.2		48.7	↑	39.7	↑	7.5			14.3	
	2011		198	159		1328		897	↑	2415		32.5		20.1		14.2		35.4	↑	40.2	↑	11.4			18.7	
H4	2006		115	242		1360		1260	↑	3160		39.6		23.5		12.1		24.2	↑	33.3		13.3			28.1	
	2010		129	214		1174		3145	↑	2984		41.2		19.7		11.5		19.8	↑	37.8		12.9			34.5	
	2012		147	119		1069		1694	↑	2531		34.5		22.4		14.6		25.4	↑	41.7	↑	10.8			37.1	
Normal range												28–56		12–35		6–41		3–18		2–38		3–20			3–39	

Abbreviations: NA, not available; ↓ or ↑, below or above the normal range, respectively.

a Normal ranges were from Stiehm RE. Immunologic Disorders in Infants and Children. 6^th^ ed. Philadelphia, PA: Philadelphia Press, 2003.

b Normal percentages were from Ref. 25.

* Memory CD4+ T cell lymphocyte percentage = CD4+CD45RO+/CD4+CD45RO+ and CD4+CD45RO−; Memory CD19+ B cell lymphocyte percentage = CD19+CD27+/CD19+CD27+ and CD19+CD27−; Activated lymphocyte percentage = CD2+HLADR+/all lymphocytes.

### Cytotoxicity to K562 Cell Lines and the Expression of Perforin and Granzyme

Lymphocyte/NK cell cytotoxicity to K562 cells was evaluated in 8 HLH episodes (8 patients) and 14 HMB episodes (4 patients) during the febrile stage (Table 4). In contrast to those with HMB episodes, the eight HLH patients, of course, met more than five out of eight diagnostic criteria including decreased cytotoxicity, which returned to a normal range during the convalescent stage among survivors.

**Table 4:** Cytotoxicity to K 562 cells and perforin and granzyme expressions in NK cells in the episodes and recovery status of EBV-HLH and HBM-CAEBV.

Patient		Cytotoxicity to K562 cell lines					Perforin expression in NK cells		Granzyme expression in NK cells	
		Effector to target cell ratio					(gated by CD56+TCRαβ− or CD16+CD56+)		(gated by CD56+TCRαβ− or CD16+CD56+)	
		25/1		12.5/1	25/1	12.5/1	Percentage (mean fluorescent intensity)		Percentage (mean fluorescent intensity)	
		Acute			Recovery		Acute	Recovery	Acute	Recovery
**EBV-associated survival**										
ES1	1995		***22.4%***	***20.8%***	42.4%	NA	NA	57.8% (48.7±19.6)	47.4% (54.4±12.4)	56.7% (61.5±18.7)
ES4	2005		***18.6%***	***17.5%***	39.7%	36.9%	***39.1% (12.6±20.0)***	NA	49.7% (58.6±13.8)	68.4% (68.4±25.8)
ES5	2006		***17.4%***	***15.6%***	31.5%	29.5%	NA	52.9% (14.2±8.7)	54.5% (63.7±22.9)	57.9% (59.8±29.2)
ES6	2006		34.9%	24.8%	36.4%	24.8%	***32.8% (34.1±18.8)***	55.7% (52.5±21.8)	44.5% (51.8±20.5)	45.2% (52.7±19.4)
**EBV-associated dead**										
EM3	2001		***16.7%***	NA	NA	NA	***25.2% (19.7±8.9)***	NA	48.3% (56.3±23.2)	NA
EM4	2002		***18.7%***	NA	NA	NA	***22.9% (31.6±12.8)***	NA	65.4% (67.4±27.8)	NA
EM5	2005		***25.9%***	NA	NA	NA	***25.2% (19.7±8.9)***	NA	44.7% (63.5±29.5)	NA
EM6	2005		***12.7%***	NA	NA	NA	***22.9% (31.6±12.8)***	NA	57.6% (42.0±17.9)	NA
**HMB-CAEBV**										
H1	2003		29.4%	24.1%	32.7%	22.8%	61.8% (58.7±27.8)	63.5% (60.4±29.5)	67.4% (42.1±24.8)	47.3% (42.4±18.5)
	2005		32.1%	NA	30.2%	NA	82.1% (74.2±35.7)	72.4% (68.5±34.7)	85.4% (68.2±34.7)	83.4% (57.7±32.7)
	2006		41.6%	32.5%	37.4%	NA	NA	NA	NA	NA
	2008		38.5%	28.5%	42.1%	NA	56.1% (54.5±19.7)	68.7% (62.5±34.7)	72.7% (67.5±33.2)	78.6% (75.1±34.4)
	2011		36.9%	NA	30.4%	NA	74.3% (64.8±31.2)	75.2% (65.4±39.2)	86.7% (64.8±39.2)	76.2% (74.0±32.7)
H2	2005		34.0%	18.4%	44.1%	33.4%	58.4% (48.2±23.4)	78.5% (68.4±27.9)	84.3% (60.4±29.5)	68.8% (60.4±29.5)
	2007		42.1%	34.2%	38.5%	NA	NA	NA	NA	NA
	2012		37.9%	28.1%	35.7%	NA	61.5% (54.1±28.2)	58.7% (64.2±34.2)	52.8% (64.3±34.2)	62.7% (70.2±32.4)
H3	2005		29.2%	27.4%	25.7%	23.8%	NA	74.8% (65.8±27.3)	79.6% (68.0±32.9)	72.4% (57.9±27.4)
	2006		28.7%	NA	27.4%	NA	56.1% (54.5±19.7)	68.7% (62.5±34.7)	72.7% (67.5±33.2)	78.6% (75.1±34.4)
	2011		34.1%	22.5%	40.8%	24.4%	74.3% (64.8±31.2)	75.2% (65.4±39.2)	86.7% (64.8±39.2)	76.2% (74.0±32.7)
H4	2006		28.7%	24.5%	32.4%	23.5%	56.4% (42.5±15.4)	52.3% (39.4±17.9)	75.1% (49.4±27.4)	68.7% (43.8±14.3)
	2010		35.2%	NA	28.2%	NA	NA	NA	NA	NA
	2012		48.7%	34.9%	34.7%	NA	75.7% (54.8±27.5)	81.5% (65.3±37.8)	68.2% (67.4±29.7)	74.8% (72.1±38.1)
Control* (n = 14)			26.1–58.9%	20.4–52.9%	26.1–58.9%	20.4–52.9%	54.7–95.2%		42.9–87.4%	

Abbreviations: NA, not available.

Bold and italicized numbers meant below the normal range.

* Healthy normal ranges were obtained from the mean ±2 standard deviations.

Intracellular perforin rather than granzyme expression significantly diminished below the normal ranges in NK cells (TCRαβ-CD56+ or CD16+56+) in the HLH episodes, but not in the HMB episodes (Table 4). The NKT (TCRαβ+CD56+) cells had similar results (data not shown). In the convalescent period, there were relatively lower perforin expressions in the four survivors of HLH despite being within the normal range. The HMB group had no such differences in cytotoxicity and in the expressions of perforin and granzyme.

### Analysis of Candidate Genes

All patients received genetic analysis for the *PFR1, Mun13-4, STX11*, *STXBP2* and *ITK* genes. Eleven male patients had further *SH2D1A/SAP* and *XIAP* sequencing but no mutation was identified.

## Discussion

Without reaching the HLH diagnostic criteria, patients with the HMB episodes could have better prognosis than those with HLH episodes. Based on an updated international meeting for EBV-associated lympho-proliferative diseases (LPD) and the recent Kimura et al. study of 108 cases [24], [26], EBV-associated LPD are defined to be overlapping umbrella syndromes and encompass five subgroups including CAEBV of T/NK-cell type (or systemic EBV plus T-cell LPD of childhood), HLH, severe mosquito bite allergy (or hypersensitivity to mosquito bite, HMB), hydroa vacciniforme (HV), and HV-like lymphoma [26]. Using these concepts to the patients here, four HMB patients initially presented as “severe mosquito bite allergy” without splenomegaly nor lymphadenopathy in the enrolled time, but gradually developed as CAEBV NK cell type with splenomegaly or/and lymphadenopathy after several HMB episodes. As noted in previous reports [26]–[30], patients with HMB have the potential to progress to HLH, and even lymphoma. To recognize the herald feature at an earlier stage by comparing HMB and HLH episodes, the HLH episodes with CD3-predominant virus load had lower perforin expression and thus related to impaired cytotoxicity that partially contributed to the development of HLH. In contrast, these HMB episodes with NK-predominant EBV virus load and higher IgE level had normal perforin expressions and did not reach a threshold to impair cytotoxicity. In HMB-CAEBV patients, higher IL-13 level, a Th2-type cytokine, were detected and could induce the differentiation of B cells and enhance a class switch to IgE [23]. Whether the majority of EBV virus shifted from NK cells in the HBM status to T-lymphocytes after several HBM episodes and cytokine alternation, reached the threshold to weaken cytotoxicity and therefore cause HLH episode remains to be determined by additional study.

The possibility of “shift” hypothesis was observed in a 35-year-old female patient who experienced at least eight HBM episodes since her first attack at the age of 21. Unfortunately, she succumbed to HLH acceleration in 1990 (beyond the study period of 1992–2012) [31]. Her EBV virus load evaluated by copy numbers from frozen PBMC revealed almost an equal amount in NK cells (10^3.4^) and in T lymphocytes (10^3.1^) during the HBM episodes, but eventually became more in T lymphocytes (10^4.2^) than NK cells (10^3.6^) in the HLH status after several HMB episodes [21], [32]. Thus in Japan, CAEBV patients with the HBM episodes are encouraged to receive hematopoietic stem cell transplantation as early as possible if suitable donors are available [24], [33], to prevent the process of HLH development and the oncogenic transformation of lymphoma [26]–[30].

Genetic defects of *PFR1, Mun13-4, STX11, STXBP2*, *ITK* (autosomal recessive), *SH2D1A/SAP*, and *XIAP* (X-linked) are responsible for hereditary HLH, with increased susceptibility to recurrent, and/or fatal EBV infection [15]–[20]. However, all are wild type in the study patients because of the conservative culture that discourage consanguineous marriage in our regions. Such findings are consistent with a recent genetic study from a cohort of 67 children of Chinese descent who had HLH and wild type candidate genes [34]. However, parallel to restored cytotoxicity after effective chemotherapy, perforin expression recovered but was maintained at the relatively lower border of the normal range. Single nucleotide polymorphism (SNP) of A91V and N252S in the *PFR1* gene was found to decrease perforin expression and function that cause atypical HLH [35], [36], but not identified of such SNP in our patients. This reflects that patients with borderline perforin expression may have decreased the perforin-granzyme B pathway to some extent, leading to insufficient apoptosis and subsequently developing HLH. EBV latent membrane protein 1 (LMP1) has been demonstrated to diminish *SH2D1A* expression and stronger inflammation [37]. Whether the similar inhibitory effect of EBV infection-associated factors (LMP1 or other antigens derived from EBV) on the perforin expression is worth being investigated further.

Notably in our patients, fatal HLH patients did not have elevated memory CD4+ cells and activated CD2+HLADR+ lymphocytes. Correlative to the differentiation and development of memory CD4+ cells and activated lymphocytes, T-cell receptor (TCR) in naïve T cells recognized MHC class II (HLA-DR) on EBV-antigen-presenting cells (APC) and triggered signaling one pathway and subsequently programmed as T effector cells after additional stimulation from signal two or more accessory pathways [38], [39]. Some subgroup of T effector cells that expressed activation molecules such as HLADR+, CD40L, ICOS, or CD69 and belonged to one pattern of the activated lymphocytes were able to continuously mature into memory T cells for robust augmentation to fight EBV infection and overcome cytokines-related catastrophic response in persistent and un-eradicated pathogen reactivation [38], [40]. Thus, elevated activated lymphocytes and memory CD4+ cells recruit more effective response to suppress EBV activation and break down the process of overwhelming HLH. In dynamics of the whole CD4+ cell pool, the amount of CD4+ effector cells going apoptosis after activation was more than those turning to memory CD4+ cells and supplemental naïve CD4+ cells. Consequently, the percentage of overall CD4+ cells trended to decrease but memory CD4+ to increase in survivors (in Table 3). However, lack of increased percentages of activated lymphocytes and memory CD4+ cells during the episode status in those fatal HLH patients implied that impending exhaustion of the whole T cell pool, challenged by EBV repeated activation, could be a warning sign of hematopoietic bone marrow failure, contributing to worse prognosis.

The attenuated perforin expression and predominant-CD3 lymphocyte EBV virus load are distinct in HLH episodes from the HMB episodes. And, the absence of elevated memory CD4+ cell or activated CD2+HALDR+ lymphocytes increase mortality in HLH episodes. Such immunologic alternation rather than genetic defects highlight the possible evolution mechanism of EBV-associated HMB episodes progressing into HLH episodes in the rare cases and warrants further verification through larger-scale studies.

## Materials and Methods

### Patients

Patients with an HMB episode had high fever, intense local erythematous responses to mosquito bites, lymphadenopathy, and splenomegaly [21], [22]. Those with fulminant HLH episode met the updated diagnostic criteria of the HLH Study Group of the Histiocyte Society [15] and all had hemophagocytosis in bone marrow aspirates. For evidence of EBV infection, EBV RT-PCR detection and viral load detected by copy numbers were determined as previously reported [41]. Serologic antibodies, including anti-viral capsid antigen IgG (EBV-VCA IgG), anti-early antigen IgG (EBEA IgG), anti-viral capsid antigen IgM (EBV-VCA IgM), and anti-nuclear antigen (EBNA) were evaluated using immuno-fluorescent ELISA.

The clinical features, treatment, prognosis, and immunologic function, including immunoglobulin levels and lymphocyte subsets of T-, B-, NK-, activated lymphocytes, and memory cells, were evaluated and compared in patients with HMB and HLH episodes after Chang Gung Human Investigation Committee approved this study and documented the humanity process. The patients’ parents or guardians provided written and verbal informed consent.

### Cytotoxicity to K562 Leukemia Cell Lines by Single-cell Level

Heparinized venous blood samples (10–15 ml) from enrolled patients and healthy controls were delivered to the laboratory within 72 hours. Peripheral blood mononuclear cells (PBMC) as effector cells were isolated from heparinized venous blood by Ficoll-Hypaque (Pharmacia Biotech, Piscataway, NJ). Effector (E) and K562 target (T) leukemia cells were added in 10 mm×10 mm wells to yield E:T ratios of 25∶1 and 12.5∶1 as indicated if there were enough cells. Control wells, including isolated target or effector cells, were assayed to determine spontaneous cell death. The cells were mixed by gentle tapping, and then centrifuged at 200×g for 1 min and incubated at 37°C in 5% CO2 over night (around 16 hours). Mouse anti-human CD45 monoclonal antibody (µl) directly conjugated with FITC (Pharmingen, San Jose, CA) were added to each tube, mixed gently, and incubated for 20 min on ice. Twenty µl of PI (Sigma, St Louis, MA) at 1 ug/ml were added to each tube before acquisition. Cytotoxicity to K562 cell lines was measured as the percentage of PI-stained K562 cells by flow cytometry, as previously described [42], [43].

### Perforin and Granzyme Expression by Flow Cytometry

Fresh PBMC (5×10^5^) were incubated for 30 min at 4°C in 50 µl of staining buffer (PharMingen) supplemented with 10% pooled human serum and 2 µl per CP-conjugated anti-TCRαβ, fluorescein isothiocyanate (FITC)-conjugated anti-CD16, and/or anti-CD56 monoclonal antibody (all from Pharmingen). The cells were then washed, pelleted, and permeabilized in Cytofix/Cytoperm solution (PharMingen) for 20 min at 4°C. The fixed or permeabilized cells were then incubated in 50 µl staining buffer with 2 µl phycoerythrin (PE)-conjugated anti-perforin and anti-granzyme mAb (PharMingen) for 30 min at 4°C. Data from three-color flow cytometry were calculated and analyzed using the cellquest software (Becton Dickinson).

### Sequence Analysis of SH2D1A/SAP, PFR1, Mun13-4, STX11, STXBP2, XIAP and ITK Genes

Total RNA was isolated from PBMC with TRIzol (GIBCOBRL, Gaithersburg, MA) as previously described [44]. Briefly, 2 ug of RNA in a total volume of 20 uL was reverse-transcribed into cDNA using oligo-dT primer and superscript RNaseH-reverse transcription (GIBCO-BRL). Two oligonucleotide primers designed from the Gene Bank were selected for each gene to cover the entire coding region of these genes, as previously described [16]–[20], [43], [44]. If a specific mutation was identified, the corresponding genomic exons/intron regions were amplified and re-confirmed.
